# Quality Analysis of Micro-Holes Made by Polymer Jetting Additive Manufacturing

**DOI:** 10.3390/polym16010032

**Published:** 2023-12-21

**Authors:** Razvan Udroiu

**Affiliations:** Department of Manufacturing Engineering, Transilvania University of Brasov, 29 Eroilor Boulevard, 500036 Brasov, Romania; udroiu.r@unitbv.ro; Tel.: +40-268-421-318

**Keywords:** micro-hole, quality analysis, dimensional accuracy, additive manufacturing, 3D printing, polymers, material jetting

## Abstract

Material jetting technology is gaining popularity, especially in polymer science, because of their high accuracy for additive manufacturing (AM) products. This paper aims to investigate the quality of micro-holes that are oriented in three basic directions, and manufactured by the material jetting AM process. This paper proposes a novel methodology to evaluate the accuracy of micro-holes features by using a transparent artifact. A test artifact with horizontal and vertical micro-holes in it, with industrial applications, was designed. Micro-holes were placed on planar and curve surfaces. Samples were manufactured by PolyJet technology from a translucent photopolymer resin which allows a facile investigation (by microscopy) of the inner structure of the micro-holes. The features of ten micro-holes printed in matte and glossy finish type, with diameters in coarse and medium options, according to ISO/ASTM 52902, were analyzed. Quality analysis of the micro-holes features was performed by microscopy investigations. The effects of main factors on the deviation of the micro-hole diameter were investigated by using the statistical design of experiments, and four control factors were considered. The best results were obtained for sample printed in matte finishing with the micro-holes oriented along the *x*-axis and *z*-axis. The smallest diameter of the micro-holes obtained by PolyJet technology on an EDEN 350 machine was 0.5 mm, but in industrial applications for a facile post-processing, a higher diameter is recommended to be used. A confirmatory experiment on a wing sample, with a number of micro-holes of the same diameter and a large length to diameter ratio of the micro-holes, was performed, and the repeatability of the results was confirmed.

## 1. Introduction

Additive manufacturing (AM), or 3D printing, is a key technology in the actual global market characterized by a grown competition, and is based on its ability to fabricate complex products directly from computer-aided design (CAD) files, and reduce the production cost and time [1]. The ISO/ASTM 52900-15 [2] standard has defined seven main types of additive manufacturing process, as follows: vat photo-polymerization (VP), binder jetting (BJ), material extrusion (ME), material jetting (MJ), sheet lamination (SL), powder bed fusion (PBF), and directed energy deposition (DED).

Material jetting is considered to be one of the most popular and mature processes used in additive plastics manufacturing [1], on the basis of its high accuracy [3,4,5], low surface roughness [6,7,8], and the good mechanical properties of the parts [9,10]. Polymer jetting (PolyJet) and multi-jet printing (MJM) are the most well-known technologies from the MJ category.

It is mentioned in [11] that the macro-sized holes have a diameter larger than 3 mm and the micro-sized holes are characterized by a diameter less than around 1 mm.

Micro-holes are made in different components, and are incorporated into electronic equipment, medical instruments, panel absorbers, micro-robots flight, microfluidic devices [12,13], and structures embedded with fiber-optic sensors. The small diameter holes were investigated in many studies by using subtractive technologies such as micro-drilling [14], electric discharge machining [15], laser drilling, ultrasonic machining, electrolytic machining, electron beam machining, fluid or abrasive jet machining, and chemical blanking. In the study [16] it was mentioned that the temperature generated in the drilling process produces the dilatations and contractions of the polymer, and it was possible to affect the surface quality.

The holes made by additive manufacturing technologies have possible applications in the accommodation of mechanical fasteners [17], surgical guides [18,19], experimental aerodynamics [20,21], and so on. In the last few years, many studies have investigated the macro-holes features and their dimensional accuracy, which have a diameter bigger than 4 mm, made by additive manufacturing [8,20,21,22,23]. The lowest dimensional errors for a matte cylindrical part at macro-scale made by PolyJet technology (Stratasys, Rehovot, Israel) were found to be *x*-axis orientation [23].

A few experimental investigations were performed on cylindrical parts at micro-scale made by material jetting. Thus, Olasek et al. [20] investigated channels with diameter of 1.5 mm and 0.4 mm that were distributed along an aerofoil, and were manufactured by MJM 3D printing technology. They concluded that quality strictly depends on the method and material chosen. On the basis of their observations, they evaluated the holes to be mostly open or blocked [20], although a dimensional deviation study was not carried out. Philipovici et al [24] conducted a non-destructive investigation of holes with PolyJet technology from Vero Black material on machine Objet Connex 350(Stratasys, Rehovot, Israel) that used X-ray Computed Tomography. They concluded that the accuracy and repeatability in the *x* and *y* axis are significantly better than in the *z*-axis, but the quality characterization of the holes was not performed. An analysis of small cylinders oriented along *z*-axis were printed in glossy finish on a Stratasys J750 3D printer (Stratasys, Rehovot, Israel), with diameters of 62.5 μm, 125 μm, 250 μm, 500 μm, 1000 μm and 2000 μm, which showed that the print quality is acceptable at 500 μm and above [25]. The ability to 3D-print different channels and orifices of dimensions smaller than 1 mm is challenging. Kara et al. [12] have demonstrated that the printed microfluidic chips with 1 mm diameter channels produced by stereolithography and fused deposition modeling are able to be used successfully to fabricate nanomedicines.

Currently, very few studies focus on micro-hole features with a diameter lower than 1 mm that are built by additive manufacturing. Studies have been conducted of the dimensional and geometrical characterization of the holes built by PolyJet technology at a macro-scale, but a lack of knowledge about the micro-scale, including micro-holes, was found. One of the root causes of the limitations of current works is a lack of information regarding the standardization of holes in additive manufacturing. Additive manufacturing standards are in continuous development, taking into account the multitude of additive manufacturing processes and materials used for this purpose. Also, the applications of micro-holes in various top fields such as microfluidic chips, experimental aerodynamics, and industry require preliminary tests before their adoption in practice.

The main aim of this paper is to identify the potential of the material jetting AM process to produce functional micro-holes in polymeric models. Its novelty lies in the attempt to perform a quality analysis of micro-holes 3D printed in different finish type from transparent polymers by using a material jetting process. In order to comprehensively assess the features of the micro-holes additively manufactured, it proposes a novel methodology that uses an artifact made of a transparent material, and an experimental design that uses optical measurements and statistical analysis. A case study focused on the additive manufacturing of a wing with a number of micro-holes or channels of the same diameter validates the proposed methodology.

## 2. Materials and Methods

**Figure 1:**
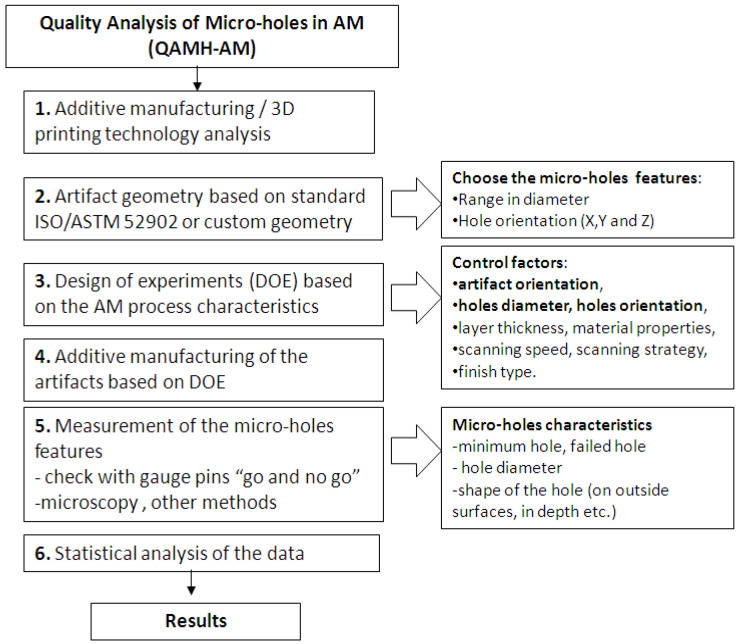
Flowchart of the proposed methodology of micro-holes investigation produced by additive manufacturing.

A new methodology related to the performance of an additive manufacturing process to produce micro-holes was proposed, as Figure 1 shows. This methodology includes the design of some specific artifacts, experiments, statistical analysis and micro-holes characterization. The methodology follows six steps as shown in Figure 1. For every additive manufacturing technology, it is important to understand the basic principles of the process and the potential parameters that influence the manufacturing process and can affect the micro-holes manufacturing.

It is recommended that the artifact geometry used to apply the methodology should be defined on the basis of standards [26]. However, a custom geometry of the artifact allows new aspects based on industrial applications of the products to be taken into consideration. The artifact size and its features influence the additive manufacturing time, material consumption, and the amount of measurement data used within the statistical analysis [27].

The experiments (DOE) were designed based on additive manufacturing process particularities by choosing the proper control factors that affect the micro-hole characteristics. The main control factors that could influence the micro-hole features are layer thickness, material properties, artifact orientation, holes orientation, and the specific finish type of the AM process, as shown in the flowchart of the proposed methodology (Figure 1). PolyJet is one of the additive manufacturing processes that deposit thin layers of 16 microns [28]. Therefore, the layer thickness used in this case is constant and does not influence the surface quality of the part.

### 2.1. Artifact Geometry

The performance of an AM process to accurately produce a specific feature should be analyzed using standard artifacts or customized models [3,29,30]. Each artifact is focused to test an aspect of the AM process, such as accuracy, resolution [29,30], or surface texture [27,31]. According to ISO/ASTM 52902,the artifacts are classified as linear artifact, circular artifact, resolution pin artifact, resolution hole artifact, resolution rib artifact, resolution slot artifact, and surface texture artifact [26].The AM-related standards do not impose a specific measurement method of artifacts features [26].

A custom test artifact with a 3D array of through micro-holes on it was designed using the SolidWorks version 2016 software (Dassault Systèmes, Waltham, MA, USA). 

**Figure 2:**
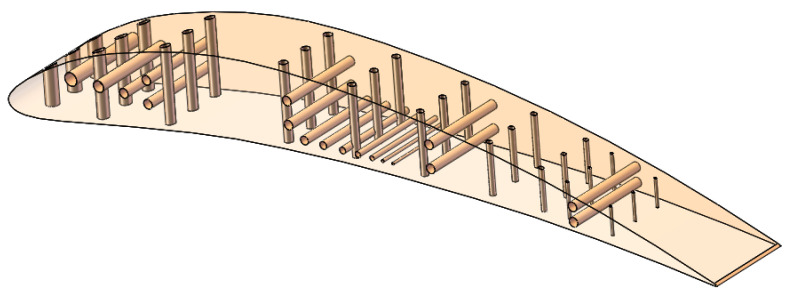
Micro-holes distribution in artifact; the artifact model is transparent.

The micro-holes distribution in artifact is presented in Figure 2. Rows of three micro-holes with the same diameters are applied on the curved surface of the artifact for all size of diameters, along the length of the artifact, as is shown in Figure 2. Also, the whole range of micro-holes was applied on a planar surface of the artifact. The holes applied on planar and curved surface make it possible to study the interaction hole-surface and test the accuracy of polymer jetting additive manufacturing technology. 

**Figure 3:**
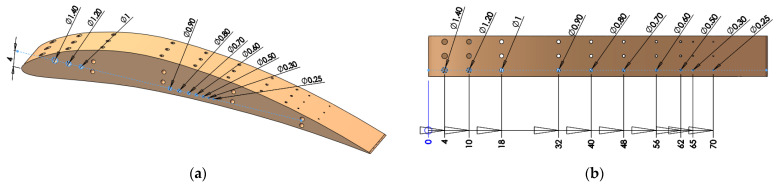
Micro-holes placed on: (**a**) planar surface; (**b**) curved surface.

The diameter range of micro-holes are chosen based on standards [26] but customized sizes are also considered based on a specific industrial application. The micro-holes were designed with diameter sizes of 0.25 mm, 0.3 mm, 0.5 mm, 0.6 mm, 0.7 mm, 0.8 mm, 0.9 mm, 1 mm, 1.2 mm, and 1.4 mm (Figure 2 and Figure 3). The maximum depth of the micro-holes was 10 mm. 

### 2.2. Design of the Experiments and Statistical Analysis

The micro-holes investigation was performed using the test artifact defined in Section 2.1. The features of ten micro-holes, 3D printed in matte and glossy finish type, with diameters in coarse and medium options (defined according to ISO/ASTM 52902), were analyzed.

The experiments were designed [32] by choosing the control factors that affect the diameter deviation (deviation) and its levels (Table 1). Diameter deviation is the difference between the nominal diameter and the measured diameter of the micro-hole. Four basic control factors that could affect micro-hole features were taken into consideration as follows, artifact orientation, finish type, micro-hole orientation and micro-hole diameter. The control factors and their levels are detailed in Table 1.

Finish type was considered as a two-level factor, namely matte and glossy finishing. This is a particular feature of the PolyJet process. The Matte finishing type applies a thin layer of support material on the whole surface of the sample. The glossy finishing type allows a relative non-uniform surface to obtain around the sample, depositing support material only on the bottom of the part, with the upper surfaces resulting in a glossy finish. The artifact orientation on the build platform is considered a two-level factor with basic orientations at 0 and 90 degrees, relative to the *x*-axis of the coordinate system. Hole orientation is considered a factor with three levels that extend along the *x*-axis, *y*-axis, and *z*-axis. Micro-hole diameter is an eight-level factor based on the diameter sizes of 0.25 mm, 0.3 mm, 0.5 mm, 0.6 mm, 0.7 mm, 0.8 mm, 0.9 mm, 1 mm, 1.2 mm, and 1.4 mm, as is shown in Table 1.

A general full factorial design with 96 factor combinations was performed to be able to investigate the influence of the control factors on the deviation of the micro-hole diameter. A statistical analysis of the data was performed to investigate and characterize the effects of control factors and their interactions on the deviation of the micro-hole diameter. Because there are more than two control factors, two-way ANOVA or generalized linear models (GLM) were used [33]. The statistical analysis was performed using Minitab 17 software (Coventry, UK), making it possible to determine the significant factors that affect the target deviation, on the basis of the indicator *p*-value and F-value. ANOVA assumptions were also checked [33]: residuals should be normally distributed, the variance of the observations in each treatment should be equal, and the response should be independent and identically distributed.

### 2.3. Manufacturing Process Specification and Quality Analysis

The samples were additive manufactured by PolyJet technology, using the Objet EDEN 350 PolyJet machine (Stratasys, Rehovot, Israel) [34]. 

**Figure 4:**
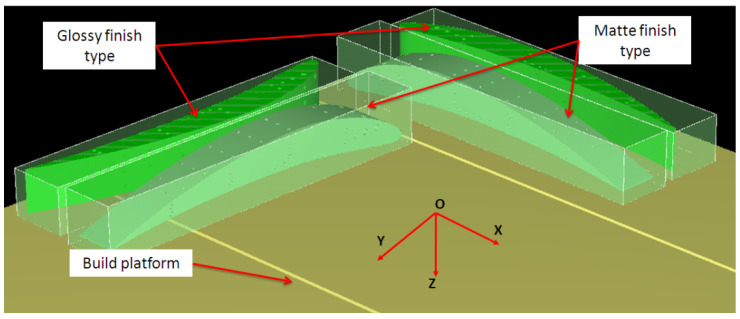
Layout of artifacts orientations on the build platform. The bounding box of samples is displayed.

The coordinate system was chosen according to [35]. The additive manufacturing process consisted of three main steps: pre-processing, processing and post-processing of the data. Objet Studio version 8.0.1.3 software (Rehovot, Israel) was used in the pre-processing step to estimate the quantity of model and support material used, and the manufacturing time. This software also manages the additive manufacturing process. Firstly, the 3D model of the test artifact was saved in STL file with conversion tolerances of 0.01 mm in deviation, and 4 degrees in angular tolerance. Then, the samples were imported in Objet Studio software, and oriented before a type of finish surface (matte or glossy) was applied for each one. The resulting layout of artifact orientations in the build platform is shown in Figure 4.

**Figure 5:**
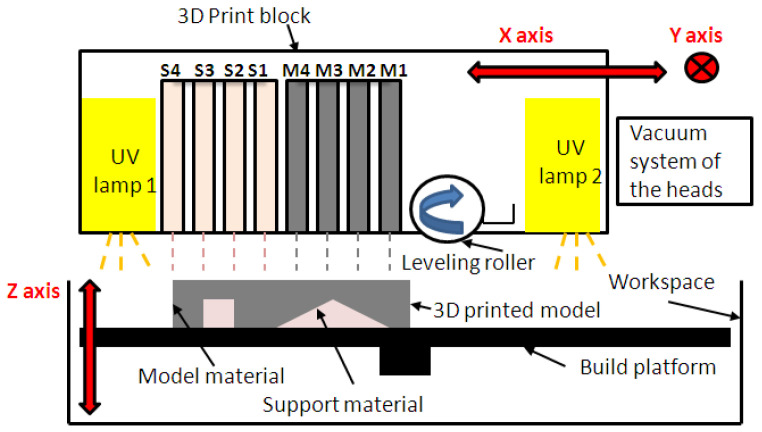
The basic principle of PolyJet technology; the 3D print block mainly contains four support inkjet heads (S1 to S4), four model inkjet heads (M1 to M4) and two UV lamps.

The additive manufacturing process on Objet EDEN 350 PolyJet machine consists of successively depositing thin layers of resins of 0.016 mm (support or model material) by using a 3D print block that moves along the *x*-axis and indexes on the *y*-axis. The layers are leveled by a roller, and then hardened (photo-polymerization) by ultraviolet (UV) light, as is shown in Figure 5.

A transparent polymeric material was chosen to manufacture the samples. This allows a deep-way investigation of the micro-hole features. Thus, FullCure 720 material as model material, with FullCure 705 as support material, supplied by Stratasys, were used to fabricate the samples. The composition of the Objet Fullcure 720 resin consists of acrylic monomer, urethane acrylate oligomer, epoxy acrylate, and photoinitiator. The main properties of Objet Fullcure 720 resin are presented in [34,36]. The support material, FullCure 705 resin, consists of acrylic monomer, polyethylene glycol 400, propane-1, 2-diol, glycerol, and photoinitiator [37,38].

The main parameters of the process were the print head temperature of 72 °C, and a print head vacuum of 6.2 atm. The manufacturing process was performed under a controlled laboratory temperature of 20 °C and a relative humidity of 30%. 

All the micro-holes of the 3D samples printed in matte finishing type were filled by support material. The horizontal micro-holes 3D printed in glossy finishing were also filled by support material. The samples needed post-processing in order to remove the support material from micro-holes. The specimens were immersed in a sodium hydroxide solution and were washed with pressurized water afterwards. Also, thin pins with smaller diameters than the micro-hole diameters were used to manually clean the support from the micro-holes. Thus, syringe needles with a 0.1 mm increment in the range diameter of 0.3 mm (codification 30G) to 1.2 mm (18G) were used to remove the support material from the micro-holes.

The investigation of micro-holes features consisted of visual inspection, checking the diameter with “go and no go” pin gauge, and microscopy measurements. Visual tests with no required equipment were performed for all four types of samples. The micro-holes were labeled according to the control factors, as is shown in Table 2.

A pin gauge size, which is considerably smaller than the nominal dimension should be, was first tested, subsequently going up in increments of 0.1 mm.

The microscopy measurements were performed by ISM-PRO software, using an ISM-PM200SB digital microscope (Insize, Boituva, Brasil) with an accuracy of 10 microns at a magnification of 150X. In order to achieve high accuracy, calibration using a calibration rule was done before the measurements. The primary measurement for the resolution hole features is the diameter of the micro-hole. The vertical holes oriented along the *z*-axis are characterized by a start hole diameter, at the lower surface and an end hole diameter at the upper surface. The horizontal holes oriented along the *x*-axis and *y*-axis are characterized by left and right end diameters of the hole and right hole diameter. The mean hole diameter was calculated based on the arithmetic mean of the hole ends.

The artifacts printed in glossy finishing are clear, transparent and allow the holes to be investigated in depth. Three measurements were performed on the deep of the clear transparent holes—on the top, middle and bottom. The mean arithmetic diameter was considered. The artifacts printed in matte finishing are not clear or transparent and the micro-holes cannot be investigated in depth. 

A series of defects that occurred during layers formation by photo-polymerization of the resin were investigated, namely the photo-polymerization affected zone (PAZ), and array affected zone (AAZ). PAZ has similar parameters to HAS (heat affected zone), and is reported in the subtractive conventional and unconventional methods of processing holes. The PAZ width parameter is defined on the basis of relation (1).

(1) $$PAZ\;width\;\left[mm\right]=\frac{\left(PAZ\;diameter\right)-(Measured\;diameter\;of\;the\;hole)}{2}$$

PAZ is actually an area adjacent to the micro-hole surface, which is affected by the photo-polymerization process in glossy finishing printing. PAZ width is only measured on the top surface. AAZ is an area around a micro-hole array.

### 2.4. Case Study as Confirmatory Experiment

**Figure 6:**
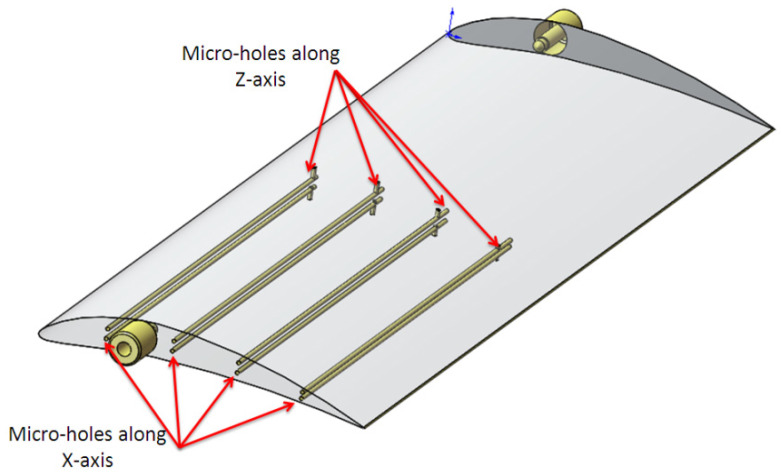
Experimental wing model that contains connected horizontal and vertical micro-holes.

An experimental wing with an array of horizontal and vertical micro-holes 1 mm in diameter was designed in SolidWorks, as is shown in Figure 6.

The horizontal micro-holes traverse the span of the wing at a depth of 120 mm, and vertical micro-holes with the same diameter are distributed on the top and bottom surface of the aerofoil. This results in a large micro-hole length-to-diameter ratio of L/D = 120.

Each horizontal micro-hole is connected with a vertical micro-hole, having applications in experimental aerodynamics. The connected micro-holes create a channel that allows the static pressure on the upper and lower surface of the wing in different locations to be captured during experimental aerodynamic tests, meaning it is possible to experimentally calculate the lift and drag forces developed on a wing at different angles of incidence.

This new design of connected holes is a solution that is practically impossible to be manufactured using any other traditional manufacturing method, such as deep drilling, than additive manufacturing. Five samples of the proposed wing were manufactured. Each sample was positioned in the optimal 3D-printing configuration, XY matte [5]. The micro-holes diameter was measured and analyzed.

## 3. Results

The layout of the four samples was manufactured in 2 h and 8 min, using 38 g of model material and 29 g of support material. 

### 3.1. Quality Analysis of the Micro-Hole Features

**Figure 7:**
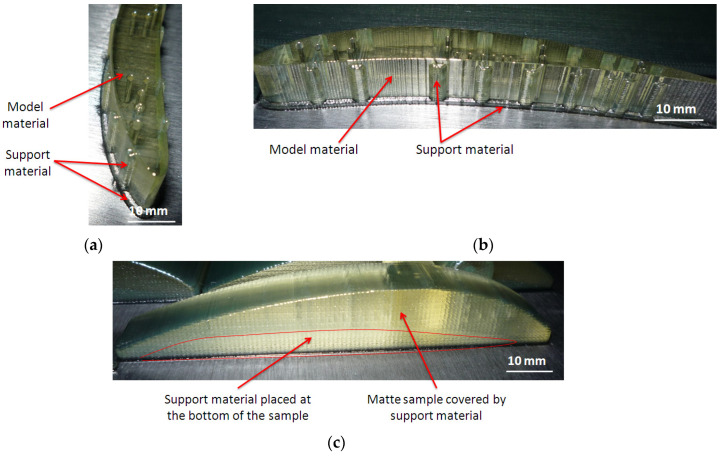
The artifacts on the build platform before post-processing: (**a**) oriented along *y*-axis, in glossy finishing (GY); (**b**) oriented along *x*-axis, in glossy finishing (GX); (**c**) oriented along *x*-axis, in matte finishing (MX)—a similar surface was obtained for a MY artifact.

Two kinds of visual inspections were performed, before and after post-processing of the samples. The artifacts placed on the build platform before post-processing are shown in Figure 7. The artifacts printed in glossy finishing present vertical striations of model material on the lateral surface, and support material deposited in the area of the micro-holes on the vertical surface. It was identified that support material of a rectangular shape was deposited on the area of the three micro-holes, as is shown in Figure 7a,b. 

The samples printed in matte finishing were covered by support material on the entire surface, as is shown in Figure 7c. Also, all the holes were filled with support material.

**Figure 8:**
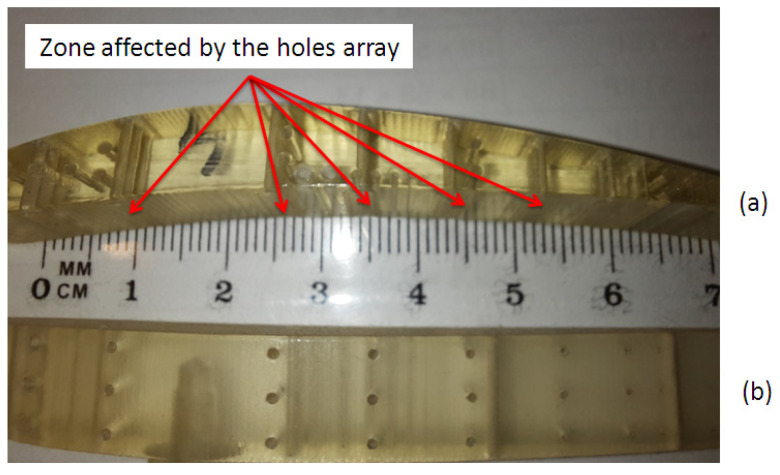
The artifacts after post-processing: (**a**) Zones affected by the three micro-holes’ array for glossy artifacts; (**b**) Smooth surface obtained for matte artifacts.

After post-processing of the samples, surface errors on the vertical walls of the glossy samples were determined at both ends of horizontal holes, as is shown in Figure 8a. These kinds of errors consist in a rectangular area around the pattern of horizontal holes. These errors were detected both on the glossy sample oriented along the *x*-axis and on the one oriented along the *y*-axis. Thus, flat areas placed around the arrays of holes were found. These types of errors are caused by support material deposited inside the horizontal holes. There were no surface defects detected on the samples printed in matte finishing type after post-processing, as is shown in Figure 8b.

A microscopy analysis was performed for quality investigation of the micro-holes for the samples GX, GY, MX, and MY.

The horizontal micro-holes oriented along the *x*-axis and *y*-axis were analyzed, and so were the vertical micro-holes along the *z*-axis. 

**Figure 9:**
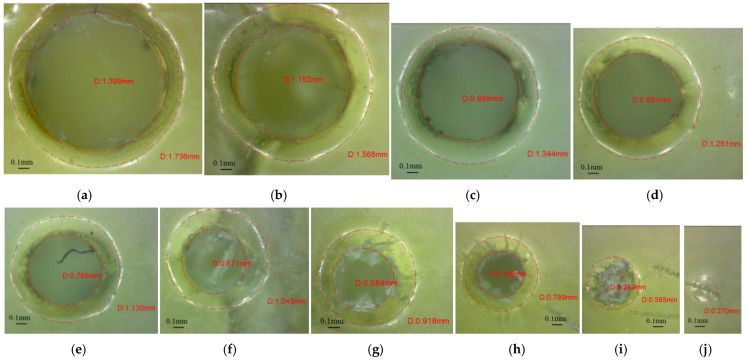
Microscopic image of the upper end of the micro-holes oriented along the *z*-axis (GX sample), which had different diameters: (**a**) 1.4 mm (D1.4_GX_Z_Up), (**b**) 1.2 mm (D1.2_GX_Z_Up), (**c**) 1 mm (D1_GX_Z_Up), (**d**) 0.9 mm (D0.9_GX_Z_Up), (**e**) 0.8 mm (D0.8_GX_Z_Up), (**f**) 0.7 mm (D0.7_GX_Z_Up), (**g**) 0.6 mm (D0.6_GX_Z_Up), (**h**) 0.5 mm (D0.9_GX_Z_Up), (**i**) 0.3 mm (D0.3_GX_Z_Up) and (**j**) 0.25 mm (D0.25_GX_Z_Up).

The upper end of the glossy micro-holes oriented along the *z*-axis is characterized by a rounded zone (PAZ), as is shown in Figure 9. The average PAZ value detected on the upper surface of the glossy micro-holes is 0.176 mm. It was detected that the glossy micro-holes of 0.25 mm in diameter were absent, as is shown in Figure 9j. A mark was present on the upper surface of these micro-holes, but the hole is absent on the lower surface. Similar morphologies for *z*-axis micro-holes orientation were found for both orientations of the glossy artifacts.

**Figure 10:**
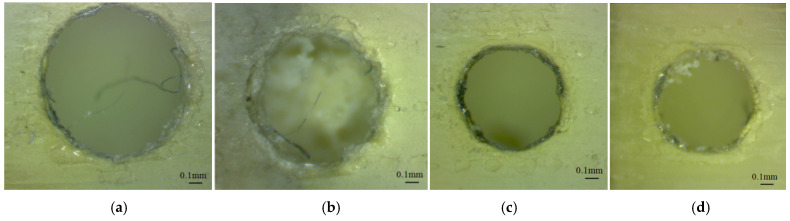

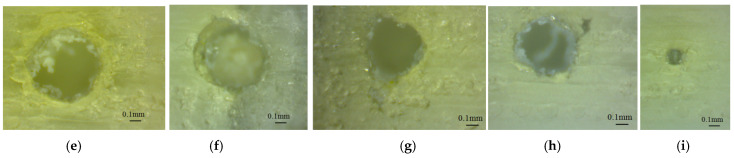
Microscopic image of the lower end of the micro-holes oriented along the *z*-axis (GX sample), with different diameters: (**a**) 1.4 mm (D1.4_GX_Z_Low), (**b**) 1.2 mm (D1.2_GX_Z_Low), (**c**) 1 mm (D1_GX_Z_Low), (**d**) 0.9 mm (D0.9_GX_Z_Low), (**e**) 0.8 mm (D0.8_GX_Z_Low), (**f**) 0.7 mm (D0.7_GX_Z_Low), (**g**) 0.6 mm (D0.6_GX_Z_Low), (**h**) 0.5 mm (D0.9_GX_Z_Low), (**i**) 0.3 mm (D0.3_GX_Z_Low).

**Figure 11:**
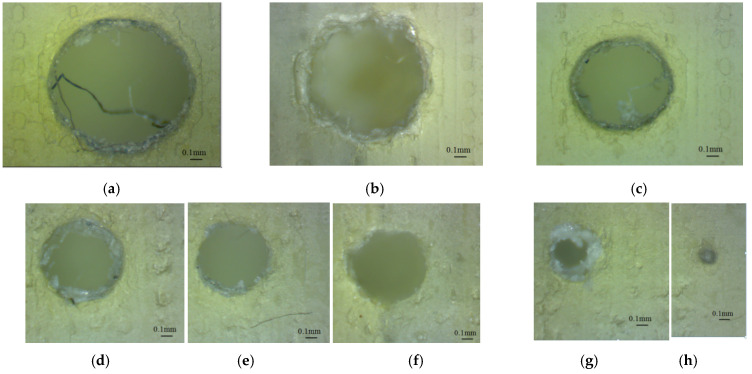
Microscopic image of the lower end of the micro-holes oriented along *z*-axis (GY sample), with different diameters: (**a**) 1.4 mm (D1.4_GY_Z_Low), (**b**) 1.2 mm (D1.2_GY_Z_Low), (**c**) 1 mm (D1_GY_Z_Low), (**d**) 0.9 mm (D0.9_GY_Z_Low), (**e**) 0.8 mm (D0.8_GY_Z_Low), (**f**) 0.7 mm (D0.7_GY_Z_Low), (**g**) 0.6 mm (D0.6_GY_Z_Low), (**h**) 0.5 mm (D0.9_GY_Z_Low).

The lower end of the glossy finishing micro-holes oriented along the *z*-axis were affected by the support material and presented a specific shape, as is shown in Figure 10 and Figure 11. Some small pieces of support material were observed in the micro-holes because the holes were not completely cleaned after the post-processing stage. The micro-holes were not perfectly round, and the measurement therefore gave the maximum inscribed diameter.

The morphology of the lower end of the glossy finishing micro-holes oriented along the *z*-axis seems to be similar for both artifact orientations, but the train of resin droplets is deposited in different directions (Figure 10 and Figure 11).

**Figure 12:**
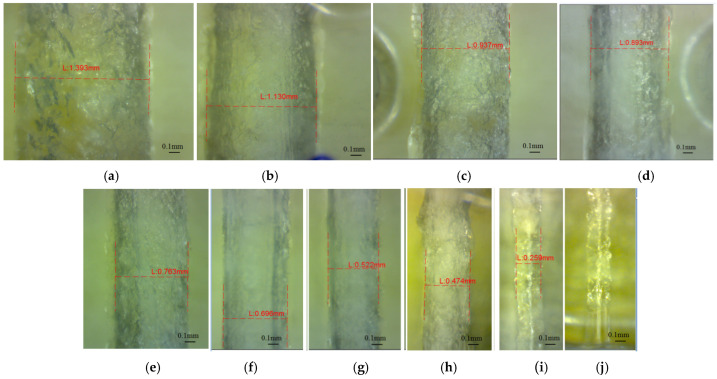
Microscopic image of the horizontal micro-holes oriented along the *y*-axis (GX sample), with different diameters: (**a**) 1.4 mm (D1.4_GX_Y), (**b**) 1.2 mm (D1.2_GX_Y), (**c**) 1 mm (D1_GX_Y), (**d**) 0.9 mm (D0.9_GX_Y), (**e**) 0.8 mm (D0.8_GX_Y), (**f**) 0.7 mm (D0.7_GX_Y), (**g**) 0.6 mm (D0.6_GX_Y), (**h**) 0.5 mm (D0.9_GX_Y), (**i**) 0.3 mm (D0.3_GX_Y), (**j**) 0.25 mm (D0.25_GX_Y).

The clear transparent upper surface of the glossy artifact makes it possible to investigate the depth horizontal micro-holes (Figure 12). The diameter of the horizontal micro-holes was measured in three sections and the mean diameter was calculated. Examples of measured diameters of the holes (from the glossy artifact printed in the X direction) are shown in Figure 12. It was determined that the horizontal micro-holes are present in the diameter range extending from 0.5 mm to 1.4 mm. The 0.3 mm diameter micro-holes were partially filled at the hole end. The 0.25 mm diameter holes were closed on the entire depth, and were filled by model material (Figure 12j). These are failed micro-holes that, even though they have a surface geometry, are solid below the surface. The lateral surface of the holes has a specific texture, as is shown in Figure 12. Similar morphologies for horizontal glossy micro-holes oriented along the *x*-axis and *y*-axis were also found.

**Figure 13:**
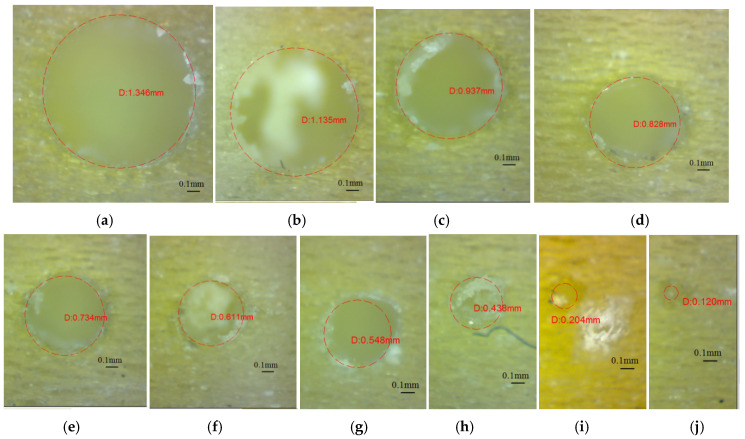
Microscopic image of the micro-holes oriented along the *x*-axis (MY sample), with different diameters: (**a**) 1.4 mm (D1.4_MY_X), (**b**) 1.2 mm (D1.2_MY_X), (**c**) 1 mm (D1_MY_X), (**d**) 0.9 mm (D0.9_MY_X), (**e**) 0.8 mm (D0.8_MY_X), (**f**) 0.7 mm (D0.7_MY_X), (**g**) 0.6 mm (D0.6_MY_X), (**h**) 0.5 mm (D0.9_MY_X), (**i**) 0.3 mm (D0.3_MY_X) and (**j**) 0.25 mm (D0.25_MY_X).

The results of the microscopy investigations for the matte samples oriented along the *x*-axis and *y*-axis showed similar morphologies, and similar characteristics were found for the left and right ends of the micro-holes. The top and bottom ends of the micro-holes also had the same characteristics. The horizontal micro-holes oriented along the *x*-axis appeared to be properly built into the diameter range of 0.3 mm to 1.4 mm, as is shown in Figure 13. The micro-holes 0.25 mm in diameter appeared to be filled, as is shown in Figure 13j. Similar results were found for the horizontal micro-holes oriented along the *y*-axis.

**Figure 14:**
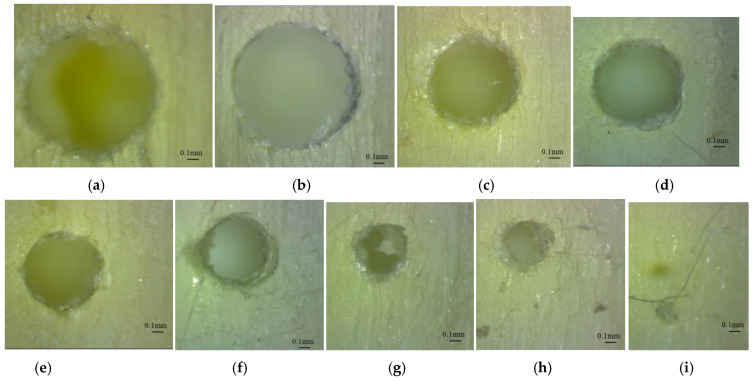
Microscopic image of the micro-holes oriented along the *z*-axis (MY sample), with different diameters: (**a**) 1.4 mm (D1.4_MY_Z), (**b**) 1.2 mm (D1.2_MY_Z), (**c**) 1 mm (D1_MY_Z), (**d**) 0.9 mm (D0.9_MY_Z), (**e**) 0.8 mm (D0.8_MY_Z), (**f**) 0.7 mm (D0.7_MY_Z), (**g**) 0.6 mm (D0.6_MY_Z), (**h**) 0.5 mm (D0.9_MY_Z), and (**i**) 0.3 mm (D0.3_MY_Z).

It was found that the matte micro-holes oriented along the *z*-axis are properly built in the diameter range that extends from 0.5 mm to 1.4 mm. The 0.3 mm and 0.25 mm diameter micro-holes were closed holes. The micro-holes oriented along the *z*-axis were determined to be irregular in shape, as is shown in Figure 14. This could be explained by the lower resolution of the printer in the X and Y directions, compared to the Z direction. 

**Figure 15:**
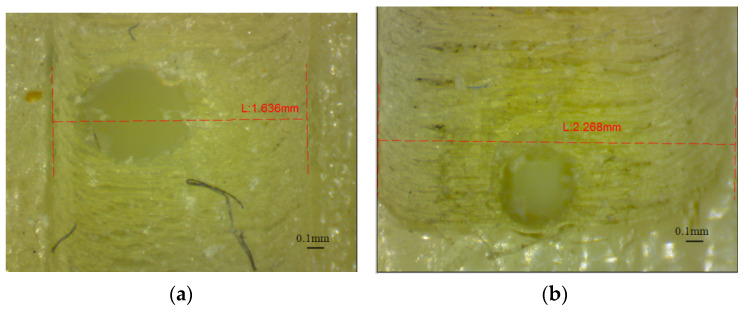
Array affected zone (AAZ) of the horizontal micro-hole, printed in glossy finishing for: (**a**) artifact oriented along the *x*-axis; (**b**) artifact oriented along the *y*-axis.

Deep investigations of the shape of the micro-holes are necessary to understand their characteristics. Thus, the rectangular area around the pattern of horizontal holes (AAZ) detected by visual investigation of the ends of the glossy micro-holes were analyzed, as is shown in Figure 15. The width of this rectangular area (array-affected zone) is in the range of 0.88 mm (D0.3 mm), and 1.786 mm (D1.4 mm) for the artifact printed in glossy mode that is oriented along the *x*-axis (Figure 15a). A relative constant width (AAZ) of around 2.26 mm was found for the artifact oriented along the *y*-axis (Figure 15b). The artifacts printed in matte finishing show a smooth surface around the array of holes oriented on the *z*-axis. No surface defects were detected in this case.

**Figure 16:**
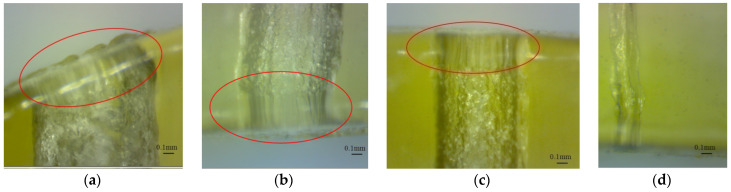
The ends of horizontal micro-holes printed in glossy finishing. The red circle shown the end area of the micro-hole: (**a**) The micro-hole end on a high sloped surface; (**b**) The micro-hole end on a low sloped surface; (**c**) The micro-hole end on a horizontal surface; (**d**) a closed micro-hole on the entire depth.

The ends of micro-holes printed in glossy mode present specific shapes which are perpendicular on the artifact surface, as is shown in Figure 16a–c. The average height of the ends is around 0.34 mm. Filled micro-hole ends are shown in Figure 16d.

**Figure 17:**
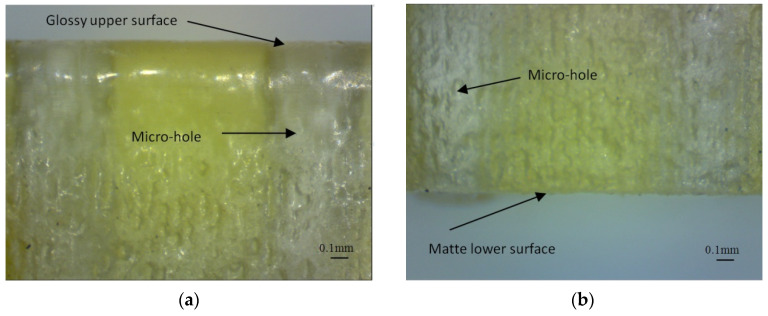
Images of the micro-holes oriented along the *z*-axis and printed in glossy finishing: (**a**) Upper surface; (**b**) Lower surface affected by the support material.

The investigation of the diameter of the depth of micro-holes oriented in the *z*-axis was difficult to perform by microscopy because of lower transparency of the lateral walls of the artifact, as is shown in Figure 17. The internal structure of the matte micro-holes is difficult to investigate based on the lower transparency of the artifact surfaces. The best transparency was obtained for the horizontal surface of the artifact printed in a glossy mode. 

### 3.2. Results of Statistical Analysis

The results of the ANOVA analysis are shown in Table 3. The hole diameter, finish type and hole orientation had a more significant influence on the diameter deviation, as long as the *p*-value was lower than the significance level of 0.001. The results were significant at the 0.1% significance level.

The artifact orientation factor had a lower percentage contribution. The most significant influence on the diameter deviation (Dev) was the hole diameter, which explained 27.98% of the total variation. The R-squared value of 95.72% indicates that the model explains all the variability of the response data around its mean. 

The interactions of hole orientation with hole diameter and artifact orientation with hole diameter had a significant effect on the diameter deviation, whereby PC% was 15.91% and 10.02%, respectively. The interactions of artifact orientation with finish type, finish type with hole orientation, and finish type with hole diameter had no significant effect on the diameter deviation (*p* > 0.05).

**Figure 18:**
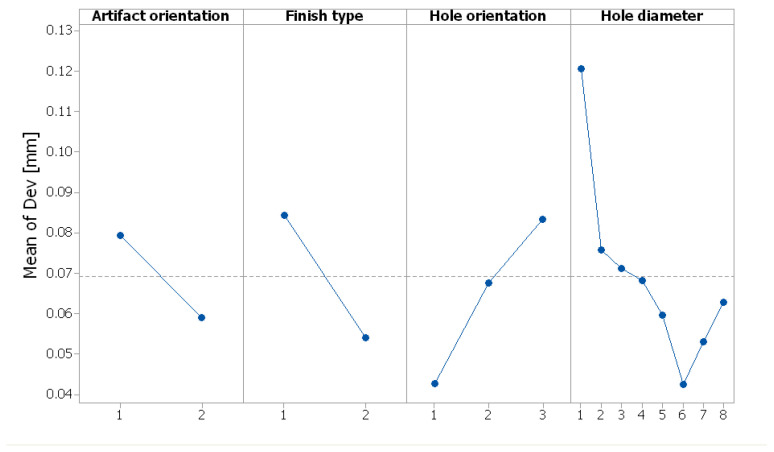
Main effects plot for diameter deviation (Dev).

**Figure 19:**
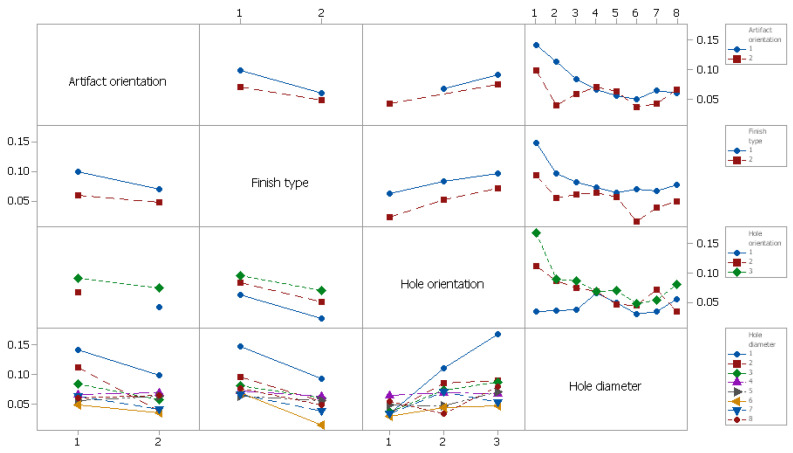
Interaction effects plot for diameter deviation (Dev).

The influence of control factors on diameter deviation was statistically evaluated using graphical methods. Thus, the following graphs were plotted: the main effects plot for deviation, interaction effects plot for deviation, and interval plot of deviation versus the control factors. The results show that artifact orientation at level 1 (0°), finish type at level 1 (matte), hole orientation at level 3 (*z*-axis), and hole diameter at level 1 (1.4 mm) had the main effects plot for deviation (Figure 18). The interaction of hole diameter factor with the other factors had a significant influence on the diameter deviation, as shown in Figure 19. 

**Figure 20:**
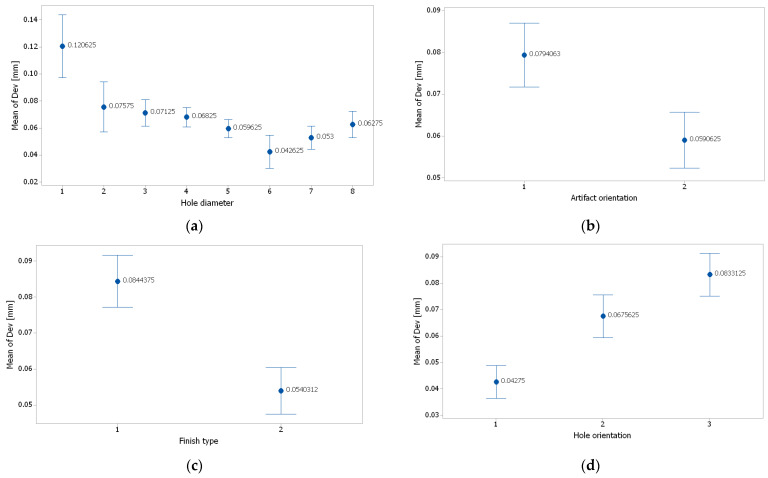
Interval plot of: (**a**) hole diameter; (**b**) artifact orientation; (**c**) finish type; and (**d**) hole orientation. Individual standard deviations were used to calculate the intervals plot. Bars are standard errors of the mean.

The interval plots with the standard error bars of each factor versus the diameter deviation (Dev) are shown in Figure 20. The difference for Dev in the hole diameter was probably not significant for the diameters of 1.2 mm, 1 mm, 0.9 mm, and 0.8 mm because all the interval bars easily overlapped (Figure 20a). Also, the hole diameter had an influence on the diameter deviation and it seems that, for hole diameter of 1.4 mm, the mean of Dev was higher, but lower for the hole diameter of 0.7 mm. 

The difference between the means for diameter deviation in artifact orientation, finish type, and hole orientation were significant because the interval bars did not overlap (Figure 20a–c). The means of Dev were lower for the artifact orientation along the *y*-axis, the glossy finish type, and the hole orientation along the *x*-axis. 

**Figure 21:**
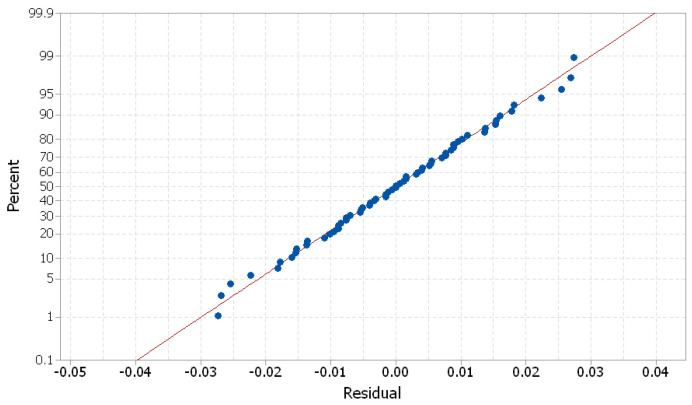
Normal probability plots of residuals for deviation (Dev).

The generalized linear model was checked for model adequacy by using the normal probability plots of residuals [27]. The residuals were normally distributed, as is shown in Figure 21.

### 3.3. Case Study as Confirmatory Experiment

**Figure 22:**
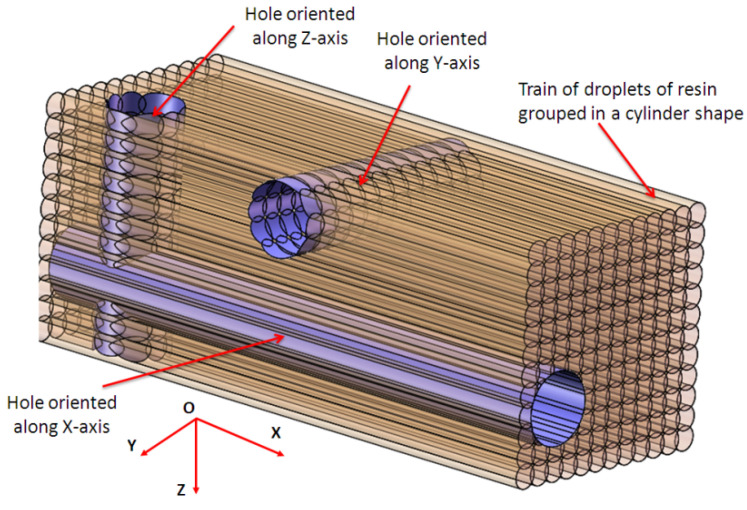
Theoretical model of the micro-holes shape.

A theoretical model of the hole shape is proposed by the author, as shown in Figure 22. The train of resin droplets grouped in cylindrical shapes, deposited by each nozzle in the X direction, forms a layer. The intersection between the micro-hole and layers forms the theoretical shape of the 3D-printed hole. The surface of holes oriented along the *y*-axis or *z*-axis results from the intersection of several cylinders from different layers, which are normal for the hole direction, resulting in a rough surface. The hole oriented along the *x*-axis consists of an intersection of the cylindrical train of droplets, which is parallel to the hole axis direction. Thus, the surface of the deep hole oriented along the *x*-axis is more homogeneous and precise than the other two types of holes. The hole surface oriented along the *x*-axis is smooth and continuous (Figure 22).

**Figure 23:**
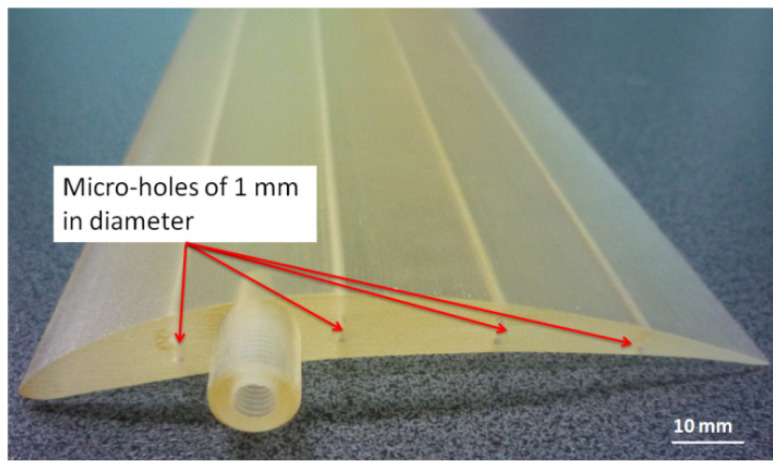
Experimental wing with eight horizontal micro-holes and eight vertical holes that are 1 mm in diameter.

Five experimental wings with eight horizontal micro-holes and eight vertical holes that were 1 mm in diameter (D1) were manufactured by PolyJet technology (Figure 23). On the basis of the previous experimental study and the theoretical model, the horizontal deep micro-holes from the wing were oriented along the *x*-axis. The manufacturing time for an experimental wing printed in matte finishing along the *x*-axis was 1 h and 24 min, and the materials consumption was 168 g of model material and 100 g of support material. 

The results of the statistical analysis of the 1 mm diameter micro-holes have shown that the coefficients of variation are lower than 10%, which confirms data heterogeneity and expresses the repeatability of the experiments, as shown in Table 4.

**Figure 24:**
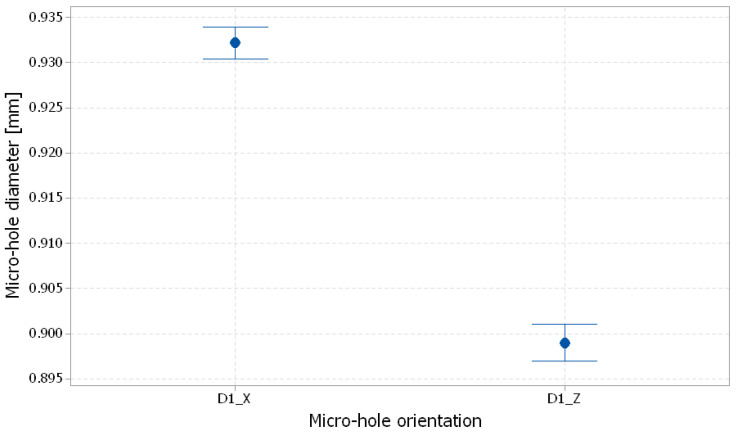
Interval plot of 1 mm diameter micro-holes (D1) for the X and Z orientations; The bars are one standard error from the mean; individual standard deviations were used to calculate the intervals.

The interval plots of the D1_X and D1_Z diameter of the micro-holes are shown in Figure 24. Individual standard deviations were used to calculate the interval plot. The highest values for the D1-measured diameter were found for the *x*-axis orientation of the wing. This was explained by the higher resolution of 0.016 mm in the *z*-axis. The smallest values of the D1-measured diameter on the *z*-axis direction (Figure 24) were caused by the lower resolution of 0.042 mm in the x-direction and y-direction. The diameter deviation along the *x*-axis is therefore smaller than the deviation along the other two axes.

## 4. Conclusions

This paper investigated the quality of micro-holes made by the material jetting AM process and characterized the features of micro-holes built in three basic directions, along the *x*-axis, *y*-axis, and *z*-axis based on the proposed methodology. The following conclusions can be drawn:

- An artifact made by translucent polymer resin allows a facile investigation (by microscopy) of the inner structure of the micro-holes.
- The experimental research demonstrated that hole diameter, finish type, and hole orientation were the most influential factors on diameter deviation of the micro-holes.
- Micro-holes are smaller than nominal. The micro-holes made by the PolyJet technology that are oriented along the *x*-axis are more accurate than the micro-holes along the *y*-axis and *z*-axis, and have an average deviation of 0.042 mm, compared with 0.067 mm and 0,083 mm, respectively.
- The experimental deviations of the diameter of the micro-holes manufactured by PolyJet technology in the glossy finish mode were lower than those obtained in the matte finish mode. After uniform surface and the quality issues of glossy mode were taken into account, the micro-holes printed in matte finishing gave the best results.
- Three situations of micro-holes construction by the AM systems can be noted, names of holes that appear properly built, holes that appear partially filled, and holes that appear absent. The smallest diameter of the properly-built micro-holes obtained by PolyJet technology using the EDEN 350 machine was 0.5 mm. After facile post-processing is taken into account, it is recommended that 1 mm diameter micro-holes should be chosen for industrial application.

The proposed methodology can be used to evaluate the accuracy of the micro-holes made by different materials, and to compare different additive manufacturing processes, in a way that considers the specific characteristics of each AM technology and chooses the proper control factors.

## Figures and Tables

**Table 1:** Control factors and their level.

Level	Target	Artifact Orientation		Finish Type		Hole Orientation		Hole Diameter	
	Symbol	Symbol	Value (°)	Symbol	Value	Symbol	Value	Symbol	Value [mm]
1	Deviation	1	0	1	Matte	1	X	1	1.4
2		2	90	2	Glossy	2	Y	2	1.2
3		-	-	-	-	3	Z	3	1
4		-	-	-	-	-	-	4	0.9
5		-	-	-	-	-	-	5	0.8
6		-	-	-	-	-	-	6	0.7
7		-	-	-	-	-	-	7	0.6
8		-	--	-	-	-	-	8	0.5

**Table 2:** Notation of 3D printed micro-holes.

Notation	Micro-Hole Diameter [mm]	Artifact Orientation and Finish Type	Micro-Hole Orientation	Position of the Micro-Hole End
Dval_GX_Z_Up	Dval	GX (X, glossy)	Z (along *z*-axis)	Up (on the upper surface)
Dval_GX_Z_Low	Dval	GX (X, glossy)	Z (along *z*-axis)	Low (on the lower surface)
Dval_GX_Y	Dval	GX (X, glossy)	Y (along *y*-axis)	-
Dval_GY_Z_Up	Dval	GY (Y, glossy)	Z (along *z*-axis)	Up (on the upper surface)
Dval_GY_Z_Low	Dval	GY (Y, glossy)	Z (along *z*-axis)	Low (on the lower surface)
Dval_GY_X	Dval	GY (Y, glossy)	X (along *x*-axis)	-
Dval_MX_Y	Dval	MX (X, matte)	Y (along *y*-axis)	-
Dval_MX_Z	Dval	MX (X, matte)	Z (along *z*-axis)	-
Dval_MY_X	Dval	MY (Y, matte)	X (along *x*-axis)	-
Dval_MY_Z	Dval	MY (Y matte)	Z (along *z*-axis)	-

**Table 3:** The percentage contribution ratio based on the generalized linear model (GLM); The symbol “*” signifies the interaction between factors.

Source	DF	Seq SS	Seq MS	*F*_exp_	*F*_0.1%_	*p*	PC (%)
Artifact orientation	1	0.006622	0.006622	19.97	17.14	0.001	6.10%
Finish type	1	0.014793	0.014793	44.61	17.14	<0.001	13.64%
Hole orientation	2	0.013004	0.006502	19.61	9.72	<0.001	11.99%
Hole diameter	7	0.030356	0.004337	13.08	7.07	<0.001	27.98%
Artifact orientation * Finish type	1	0.001131	0.001131	3.41	17.14	0.086	1.04%
Artifact orientation * Hole diameter	7	0.010874	0.001553	4.69	7.07	0.007	10.02%
Finish type * Hole orientation	2	0.00267	0.001335	4.03	9.72	0.042	2.46%
Finish type * Hole diameter	7	0.004573	0.000653	1.97	7.07	0.133	4.22%
Hole orientation * Hole diameter	14	0.017266	0.001233	3.72	5.92	0.01	15.91%
Artifact orientation * Finish type * Hole diameter	7	0.002562	0.000366	1.1	7.07	0.412	2.36%
Error	14	0.004642	0.000332				4.28%
Total	63	0.108491					100.00%

**Table 4:** Statistics of 1 mm diameter micro-holes.

Micro-Hole Orientation	Mean Value [mm]	Standard Deviation [mm]	Coefficient of Variation [%]
X	0.932	0.01129	1.21
Z	0.899	0.01274	1.42

## Data Availability

Data are contained within the article.
